# Network Pharmacology and Molecular Docking-Based Investigation of Empagliflozin’s Therapeutic Potential in Chronic Kidney Disease

**DOI:** 10.3390/life16050719

**Published:** 2026-04-23

**Authors:** Aman Tedasen, Moragot Chatatikun, Ratana Netphakdee, Jason C. Huang, Atthaphong Phongphithakchai

**Affiliations:** 1 School of Allied Health Sciences, Walailak University, Nakhon Si Thammarat 80161, Thailand; aman.te@wu.ac.th (A.T.); moragot.ch@wu.ac.th (M.C.); ratana.ne@mail.wu.ac.th (R.N.); 2 Research Excellence Center for Innovation and Health Products, Walailak University, Nakhon Si Thammarat 80161, Thailand; 3 Department of Biotechnology and Laboratory Science in Medicine, National Yang Ming Chiao Tung University, Taipei 112304, Taiwan; jasonhuang@nycu.edu.tw; 4 Nephrology Unit, Division of Internal Medicine, Faculty of Medicine, Prince of Songkhla University, Songkhla 90110, Thailand

**Keywords:** chronic kidney disease, empagliflozin, ADMET, network pharmacology, molecular docking

## Abstract

Chronic kidney disease (CKD) is a progressive global health challenge. While empagliflozin, a selective SGLT2 inhibitor, is known to attenuate CKD progression through mechanisms beyond glycemic control, the precise molecular pathways remain incompletely characterized and warrant further investigation. This study employed an integrated network pharmacology and molecular docking approach to elucidate the multi-target mechanisms of empagliflozin in CKD. Initial evaluation demonstrated that empagliflozin exhibits favorable physicochemical properties, drug-likeness, and ADMET profiles, supporting its potential as an effective orally administered therapeutic option for CKD management. Network analysis identified 221 shared molecular targets between empagliflozin and CKD-associated genes. Topological analysis of the protein–protein interaction (PPI) network revealed ten critical hub proteins—GAPDH, IL6, EGFR, HSP90AA1, NFKB1, HSP90AB1, MTOR, MAPK3, IL2, and PIK3CA—which serve as key regulators in CKD pathophysiology. Gene Ontology and KEGG pathway enrichment analyses indicated that these shared targets are significantly involved in phosphorylation, signal transduction, and central signaling cascades associated with CKD progression, including the PI3K-Akt, FoxO, HIF-1, and AGE-RAGE pathways. Molecular docking simulations corroborated empagliflozin’s multi-target affinity, demonstrating particularly strong binding energies toward HSP90AB1 (−10.85 kcal/mol), MAPK3 (−9.46 kcal/mol), and EGFR (−9.38 kcal/mol). Empagliflozin maintained stable hydrogen bonding throughout the 200-ns molecular dynamics simulation, primarily with GLN18, GLU42, SER45, ASN46, ASN101, GLY130, and TYR134, underscoring its persistent and well-anchored interaction with HSP90AB1. Collectively, these findings provide crucial mechanistic insights, suggesting that empagliflozin might exerts therapeutic effects by modulating interconnected pathways regulating inflammation, oxidative stress, and metabolic homeostasis, thereby reinforcing its role as a comprehensive, multi-target therapeutic strategy for CKD management. Nonetheless, validation through in vitro experiments remains necessary.

## 1. Introduction

Chronic kidney disease (CKD) affected approximately 697.5 million people worldwide by 2017, about 9.5 percent prevalence across 162 countries, and is projected to cause 4 million deaths by 2040 [1]. CKD typically arises from glomerular hypertension, tubulointerstitial fibrosis, and chronic inflammation, leading to a gradual decline in kidney function and eventual end-stage renal disease (ESRD) [2]. Diabetes mellitus (DM), expected to affect 783.2 million individuals by 2045, contributes to nephropathy in 20–40% of patients and already causes kidney injury in roughly 5% of newly diagnosed type 2 cases, thereby greatly compounding the risk of CKD [3]. Among the 463 million adults living with diabetes, 30–40% develop diabetic kidney disease (DKD), which begins with microalbuminuria and progresses through proteinuria, declining glomerular filtration, chronic kidney failure, and ultimately death [4,5]. In the diabetic milieu, hyperglycemia—exacerbated by altered renal hemodynamics, dyslipidemia, and excessive renal glucose reabsorption (accounting for nearly half of systemic glucose turnover)—triggers inflammatory cascades via advanced glycation end products and protein kinase C pathways [6]. These processes thicken the glomerular basement membrane, expand the mesangial matrix, and induce glomerulosclerosis. Hyperglycemia also upregulates transforming growth factor-β (TGF-β), the master profibrotic cytokine, driving unchecked extracellular matrix and collagen deposition, a response further intensified by angiotensin II and AGEs [7]. Renal fibrosis, the common final pathway, is characterized by relentless expansion of interstitial fibroblasts and deposition of collagen I and III, which obliterate normal tubulointerstitial architecture. Epithelial–mesenchymal transition further activates α-smooth muscle actin–expressing myofibroblasts, perpetuating fibrotic remodeling [8]. Concurrently, elevated pro-inflammatory cytokines such as interleukin-6 (IL-6) and tumor necrosis factor-α (TNF-α), together with oxidative stress and mitochondrial dysfunction, amplify tissue injury [9]. Despite current treatments, including glycemic control, dialysis, transplantation, and various hypoglycemic agents, many patients continue to suffer irreversible fibrosis, high morbidity and mortality, adverse events such as hypoglycemia, lactic acidosis, and gastrointestinal damage, while also facing substantial financial burdens [10,11].

**Figure 1:**
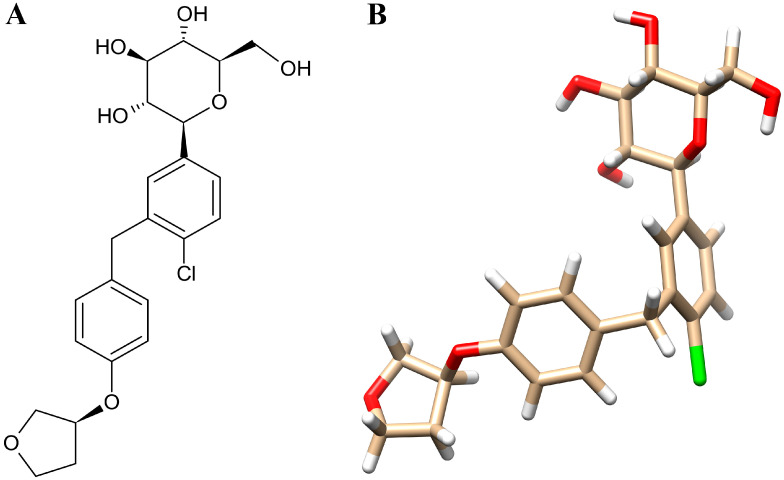
Chemical structure of empagliflozin in 2D (**A**) and 3D (**B**).

Empagliflozin is a selective sodium–glucose co-transporter 2 (SGLT2) inhibitor that reduces renal glucose reabsorption in the proximal tubule [12]. The two-dimensional and three-dimensional chemical structures of empagliflozin are shown in Figure 1. By promoting glycosuria, it lowers both fasting and postprandial blood glucose levels while modestly reducing intraglomerular pressure, thereby providing combined metabolic and hemodynamic kidney protection [13]. Beyond its glucose-lowering effects, empagliflozin attenuates CKD progression through multiple complementary mechanisms. It enhances tubuloglomerular feedback to alleviate intraglomerular hypertension, reduces albuminuria by approximately 30–40%, and mitigates renal fibrosis and inflammation [14,15]. By inhibiting SGLT2 in the proximal tubule, empagliflozin increases sodium delivery to the macula densa, restoring tubule-glomerular feedback and inducing afferent arteriolar constriction. This process reduces intraglomerular pressure and limits glomerular hyperfiltration, a key contributor to CKD progression [16]. In addition to its hemodynamic benefits, empagliflozin exerts favorable cellular effects. By promoting glycosuria, it lowers plasma glucose and hemoglobin A1c levels, thereby reducing glucotoxicity in renal tubular cells. Moreover, empagliflozin downregulates pro-inflammatory cytokines such as IL-6 and TNF-α, as well as profibrotic mediators including TGF-β, resulting in decreased oxidative stress, inhibition of myofibroblast activation, and reduced extracellular matrix deposition [17]. Collectively, these mechanisms underscore empagliflozin’s multifaceted renoprotective effects, encompassing metabolic, hemodynamic, and anti-inflammatory pathways. Despite evidence of empagliflozin’s renoprotective effects, the precise molecular mechanisms underlying these benefits remain incompletely understood. Most existing in vivo and mechanistic studies adopt reductionist approaches, focusing on isolated pathways rather than the broader network of interactions driving CKD pathogenesis. This limitation underscores the need for system-level strategies capable of capturing multi-target drug actions. Network pharmacology provides such a framework, offering a holistic perspective to bridge these gaps and uncover novel mechanistic insights.

Network pharmacology, first articulated by Hopkins in 2007, has transformed drug discovery by shifting from a one-drug–one-target paradigm to a systems-level, multi-target strategy [18]. By integrating omics technologies, systems biology, bioinformatics, computational modeling, and artificial intelligence, it constructs multilayered drug–target–disease networks that capture the full complexity of molecular interactions [19]. These networks accelerate target identification, enable drug repurposing, and reveal synergistic mechanisms of multi-component or polyherbal therapies [20]. Unlike traditional pharmacology’s narrow focus on single targets, network pharmacology provides a holistic view of disease pathogenesis and treatment, mapping drugs or phytochemicals to their targets, pathways, and clinical phenotypes [21]. By combining systems biology with pharmacological data, this approach facilitates a comprehensive examination of how drugs interact with multiple molecular targets and biological pathways associated with disease progression [22]. Through network pharmacology, researchers can systematically map complex interactions between pharmacological agents, target proteins, and pathophysiological processes [23]. This enables the identification of both established and previously unrecognized molecular mechanisms, thereby uncovering new opportunities for therapeutic intervention. When applied to empagliflozin, this methodology reveals its capacity to exert effects beyond glycemic control by acting on multiple targets implicated in CKD pathogenesis. Moreover, network-based analysis can highlight novel targets potentially amenable to pharmacological modulation, expanding the therapeutic horizon of empagliflozin within, and possibly beyond, the context of CKD.

## 2. Materials and Methods

### 2.1. Prediction of Chemo-Informatics, Drug Likeness and ADMET

The physicochemical properties of empagliflozin, including its lipophilicity, aqueous solubility, and overall drug-likeness, were systematically assessed in relation to Lipinski’s Rule of Five (RO5), a widely recognized guideline for predicting the oral bioavailability of small-molecule therapeutics. To obtain a comprehensive profile, key molecular descriptors, including molecular weight, partition coefficient (LogP), topological polar surface area (TPSA), number of rotatable bonds, hydrogen bond acceptors (HBA), and hydrogen bond donors (HBD), were calculated using the SwissADME web tool (http://www.swissadme.ch; accessed on 24 April 2025) [24]. These descriptors provide critical insights into the compound’s permeability, solubility, and likelihood of successful oral absorption.

In addition to physicochemical profiling, the pharmacokinetic behavior of empagliflozin was modeled using in silico ADMET (Absorption, Distribution, Metabolism, Excretion, and Toxicity) predictions. For this purpose, the compound’s SMILES notation was submitted to the pkCSM platform (https://biosig.lab.uq.edu.au/pkcsm/prediction; accessed on 24 April 2025), which employs graph-based signatures to estimate a wide range of pharmacokinetic and toxicity parameters [25]. Although empagliflozin is already an established orally approved therapeutic, the combined use of SwissADME for physicochemical and drug-likeness profiling together with pkCSM for ADMET prediction provides valuable complementary insights. This dual in silico approach not only corroborates its known pharmacokinetic suitability but also offers a systematic framework for benchmarking against related compounds and guiding future drug design efforts.

### 2.2. Molecular Targets of Empagliflozin in Chronic Kidney Disease

Empagliflozin’s chemical structure was retrieved from the PubChem database (https://pubchem.ncbi.nlm.nih.gov/) as a canonical SMILES string. This SMILES notation was submitted to the SwissTargetPrediction platform (http://www.swisstargetpredic-tion.ch; accessed on 24 April 2025) to forecast potential human protein targets using default parameters. The initial list of predicted targets was then mapped to UniProt entries (https://www.uniprot.org/; accessed on 24 April 2025) to ensure annotation consistency, remove duplicates, and standardize gene and protein names [26]. A catalog of chronic kidney disease–associated genes was assembled by querying the GeneCards database (https://www.genecards.org/; accessed on 24 April 2025) with the term “chronic kidney disease”. The curated empagliflozin targets were compared against this CKD gene set using the Venn diagram tool from Bioinformatics & Evolutionary Genomics (https://bioinformatics.psb.ugent.be/webtools/Venn/; accessed on 24 April 2025). The overlapping entries from this analysis represent a refined panel of candidate molecular targets for empagliflozin in CKD therapy [27].

### 2.3. Protein-Protein Interaction (PPI) Network Construction

To explore the functional relationships among the shared targets of empagliflozin and CKD, a PPI network was constructed using STRING version 12.0 (species: *Homo sapiens*), with a confidence score threshold of 0.40 applied to retain medium-confidence associations (http://string-db.org/; accessed on 24 April 2025) [28]. The resulting dataset, consisting of proteins as nodes and predicted interactions as edges, was exported in TSV and PNG formats and subsequently imported into Cytoscape version 3.10.1 for visualization and topological analysis (https://cytoscape.org/). Network properties, including node degree, clustering coefficient, and betweenness centrality, were quantified using the Network Analyzer tool to characterize the overall architecture. To identify key regulators, CytoHubba was employed to rank proteins based on degree centrality (https://apps.cytoscape.org/apps/cytohubba; accessed on 24 April 2025). The top ten nodes were designated as hub targets, representing potential critical proteins for further experimental validation [29].

### 2.4. Gene Ontology (GO) and Kyoto Encyclopedia of Genes and Genomes (KEGG) Enrichment Analysis

To systematically investigate the biological significance of the shared targets between empagliflozin and CKD, Gene Ontology (GO) and Kyoto Encyclopedia of Genes and Genomes (KEGG) enrichment analyses were performed using ShinyGO version 0.85 (http://bioinformatics.sdstate.edu/go/; accessed on 24 April 2025). These analyses were designed to elucidate both the functional roles and signaling pathways associated with the overlapping targets in *Homo sapiens*. Statistical significance was determined using a false discovery rate (FDR) threshold of ≤0.05 (*p* < 0.05). From a pool of up to 2000 candidate terms, the top 20 enriched categories were selected for both GO and KEGG analyses to ensure focus on the most biologically relevant results. For GO enrichment, results were organized into the three principal domains: Biological Process (BP), Cellular Component (CC), and Molecular Function (MF). These terms were visualized as plots ranked by −log10(FDR), enabling a comparative assessment of how empagliflozin may influence cellular processes, subcellular localization, and molecular activities [30]. In parallel, KEGG pathway enrichment was conducted under identical statistical thresholds to identify key signaling cascades implicated in CKD pathogenesis and potentially modulated by empagliflozin [31]. Together, these enrichment analyses provide a comprehensive functional map of the shared targets, highlighting pathways that drive disease phenotypes and offering mechanistic insights into empagliflozin’s therapeutic relevance beyond glycemic control. By integrating GO and KEGG results, this study underscores the complex interplay among molecular players, signaling networks, and disease mechanisms, thereby laying the groundwork for hypothesis generation and future experimental validation.

### 2.5. Molecular Docking Software Tools and Ligand Preparation

Molecular docking simulations were performed using an integrated workflow of computational platforms, including UCSF Chimera (version 10.17.3), AutoDock Tools (ADT, version 4.2.6), BIOVIA Discovery Studio 2024, the RCSB Protein Data Bank (https://www.rcsb.org/; accessed on 28 April 2025), and the PubChem database (https://pubchem.ncbi.nlm.nih.gov/; accessed on 28 April 2025). The 3D structure of the ligand, empagliflozin, was retrieved from PubChem in SDF format (PubChem CID_11949646) and subjected to energy minimization in UCSF Chimera using Steepest Descent and Conjugate Gradient algorithms. Optimization was conducted under the AMBER force field with AM1-BCC charge assignment, and hydrogen atoms were added at physiological pH (7.4) to ensure accurate protonation states. Bond orders, torsional angles, and topology were refined to improve structural reliability, after which the ligand was converted into Auto-Dock-compatible formats (PDB and PDBQT) [32].

### 2.6. Target Preparation and Docking Procedure

Crystal structures of key CKD-related proteins—including GAPDH (6M61), IL6 (1ALU), EGFR (8A27), HSP90AA1 (5H22), NFKB1 (1SVC), HSP90AB1 (6N8Y), MTOR (3TL5), MAPK3 (4QTB), IL2 (1PW6), and PIK3CA (5XGH), were retrieved from the RCSB Protein Data Bank, all with resolutions below 3 Å to ensure structural accuracy. Using BIOVIA Discovery Studio, non-essential water molecules and co-crystallized ligands were removed, and the cleaned structures were saved in PDB format before conversion to PDBQT with ADT. Docking simulations were conducted in AutoDock4 (version 4.2.6) using the Lamarckian Genetic Algorithm with 50 GA runs and a population size of 200, while grid maps were generated with Autogrid 4.2. Binding affinities and conformations were analyzed, and molecular interactions were visualized in BIOVIA Discovery Studio [33]. Final docking results were evaluated based on binding energies, interacting residues, and chemical bonding patterns, providing a comprehensive assessment of empagliflozin’s binding potential against CKD-related targets.

To validate the docking protocol, co-crystallized ligands from the three-dimensional structures of the target proteins were prepared and re-docked using the same computational procedures applied to the test compounds. The resulting binding poses were compared with their experimentally determined crystallographic conformations to assess the accuracy of the docking parameters. In cases where co-crystallized ligands were unavailable, well-established inhibitors or reference ligands reported in the literature were used as substitutes to ensure reliable benchmarking. This combined strategy confirmed that the docking workflow could accurately reproduce native binding orientations and provided confidence in the robustness of the protocol for subsequent ligand evaluation.

### 2.7. Molecular Dynamics Simulation

Molecular dynamics (MD) simulations were employed to investigate the time-dependent behavior of CKD–empagliflozin complexes, providing insights into ligand-induced effects on protein flexibility and binding. Complexes were prepared at pH 7.0 using the Protein Preparation Wizard (Schrödinger Release 2022-3: Maestro, Schrödinger, LLC, New York, NY, USA) Protein Preparation Wizard, which added hydrogens, assigned bond orders, rebuilt missing side chains and loops, optimized hydrogen-bond networks, and sampled water orientations, followed by application of the OPLS4 force field. Each system was solvated in a 10 Å × 10 Å × 10 Å orthorhombic TIP3P water box, neutralized with 0.15 M Na^+^ and Cl^−^ ions, and parameterized for simulation. Production runs were conducted for 200 ns under an NPT ensemble at 310 K and 1.01 bar, with long-range electrostatics treated using the Smooth Particle Mesh Ewald (PME) method and solvent modeled by a simple point-charge representation. Trajectory analyses, performed via the Simulation Interaction Diagram Wizard, included RMSD profiles, RMSF plots, ligand–protein contact maps, and timeline interaction analyses. All simulations and analyses were carried out using Desmond (Schrödinger Release 2022-3: Maestro, Schrödinger, LLC, New York, NY, USA), providing a comprehensive view of structural stability, conformational dynamics, and key interaction hotspots throughout the simulation [33].

## 3. Results

### 3.1. Drug-Likeness Prediction of Empagliflozin

The physicochemical and drug-likeness properties of empagliflozin were evaluated using SwissADME tools (Table 1). Empagliflozin has a molecular formula of C_23_H_27_ClO_7_ and a molecular weight of 450.91 Da, which falls within the acceptable range for oral drugs (<500 Da). The compound contains 31 heavy atoms, including 12 aromatic atoms, and exhibits a balanced fraction Csp^3^ (0.48), suggesting an appropriate mix of aromaticity and saturation that supports structural diversity and favorable pharmacokinetics. With 6 rotatable bonds, 7 hydrogen bond acceptors, and 4 hydrogen bond donors, empagliflozin meets the flexibility and polarity requirements for oral bioavailability. Its topological polar surface area (TPSA) of 108.61 Å^2^ is slightly above the 90 Å^2^ threshold often associated with optimal intestinal absorption but remains within the acceptable range (<140 Å^2^), indicating adequate permeability. Lipophilicity predictions across multiple models (iLOGP, XLOGP3, WLOGP, MLOGP, SILICOS-IT) yielded values between 0.70 and 3.18, with a consensus LogP of 1.97, reflecting moderate lipophilicity. This balance suggests that empagliflozin is sufficiently hydrophobic to cross lipid membranes while retaining solubility. Water solubility predictions classified the compound as soluble to moderately soluble. The ESOL model indicated a solubility of 7.06 × 10^−2^ mg/mL (Log S −3.80), while the Ali model predicted 5.19 × 10^−2^ mg/mL (Log S −3.94). In contrast, SILICOS-IT predicted slightly lower solubility at 2.54 × 10^−2^ mg/mL (Log S −4.25), classifying the compound as moderately soluble. These values suggest that empagliflozin has acceptable aqueous solubility for oral administration, although formulation strategies may further enhance dissolution. In terms of drug-likeness filters, empagliflozin complies with Lipinski’s Rule of Five, Ghose, Veber, Egan, and Muegge criteria, confirming its suitability as an orally active drug candidate. The bioavailability score of 0.55 indicates a moderate probability of achieving systemic exposure after oral dosing, consistent with its known clinical pharmacokinetics.

### 3.2. Pharmacokinetic and ADMET Characteristics Prediction of Empagliflozin

The pkCSM predictions indicate that empagliflozin possesses a generally favorable pharmacokinetic profile for oral administration (Table 2). Although empagliflozin demonstrates moderate intestinal absorption (55.9%) and predicted solubility, these properties are sufficient to achieve therapeutic efficacy, as evidenced by its established role in clinical practice. The compound is both a substrate and inhibitor of P-glycoprotein, which may influence efflux and drug–drug interactions, but it shows no significant inhibition of major CYP isoforms, thereby reducing the risk of metabolic interactions. Empagliflozin is primarily metabolized by CYP3A4, with moderate clearance (0.404 log mL/min/kg), supporting once-daily dosing. Distribution predictions indicate low to moderate tissue penetration (VDss −0.387 log L/kg) and high plasma protein binding (92.7%), while negligible BBB and CNS permeability values confirm its peripheral selectivity, minimizing central nervous system side effects.

The toxicity profile is also favorable, with no predicted hepatotoxicity, AMES mutagenicity, or skin sensitization. Acute and chronic rodent toxicity values suggest acceptable safety margins. The absence of hERG I inhibition reduces the likelihood of severe cardiotoxicity, although a flagged hERG II interaction warrants monitoring. Overall, empagliflozin demonstrates a balanced ADMET profile characterized by moderate oral absorption, predictable metabolism, limited CNS penetration, and low toxicity risks. These properties support its role as an orally active agent in diabetes and CKD management, while highlighting P-gp interactions and hERG II liability as important considerations for clinical monitoring.

### 3.3. Target Proteins Identification and Analysis

**Figure 2:**
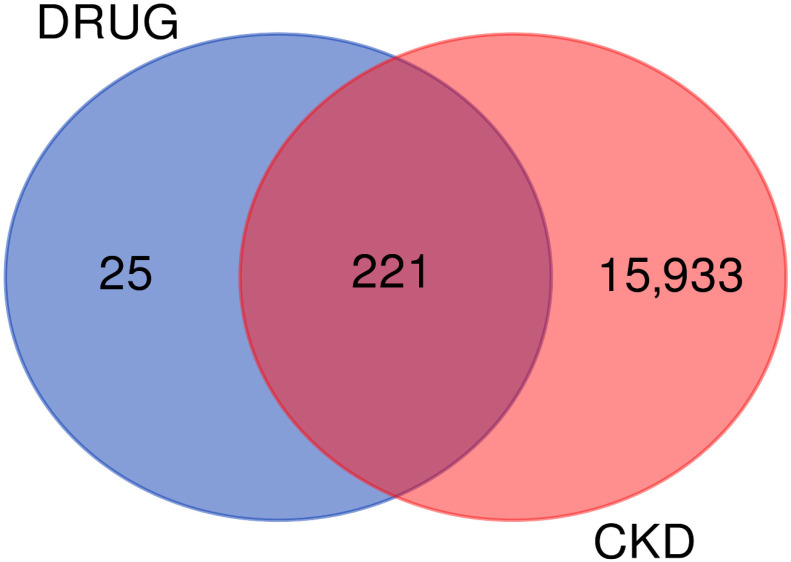
Identification of shared targets between empagliflozin and CKD through comparative integration of drug target databases and CKD-related gene sets. The Venn diagram illustrates the overlap between predicted drug targets of empagliflozin (blue circle) and CKD-associated genes (red circle). A total of 25 targets were unique to empagliflozin, while 15,933 genes were specific to CKD. Importantly, 221 targets were identified at the intersection, representing potential shared molecular mediators.

The integration of drug target databases with CKD-related gene sets revealed a notable overlap between empagliflozin targets and CKD-associated genes. Using the SwissTargetPrediction database, 246 potential molecular targets of empagliflozin were identified, while 16,154 CKD-related genes were retrieved from the GeneCards database. As illustrated in Figure 2, 25 targets were unique to empagliflozin and 15,933 were specific to CKD, whereas 221 targets were shared between the two datasets. These overlapping targets represent key molecular mediators that may link the pharmacological actions of empagliflozin to CKD pathophysiology. Importantly, this shared subset provides a focused pool of candidates for functional characterization and further experimental validation.

### 3.4. Results of PPI Network Construction

**Figure 3:**
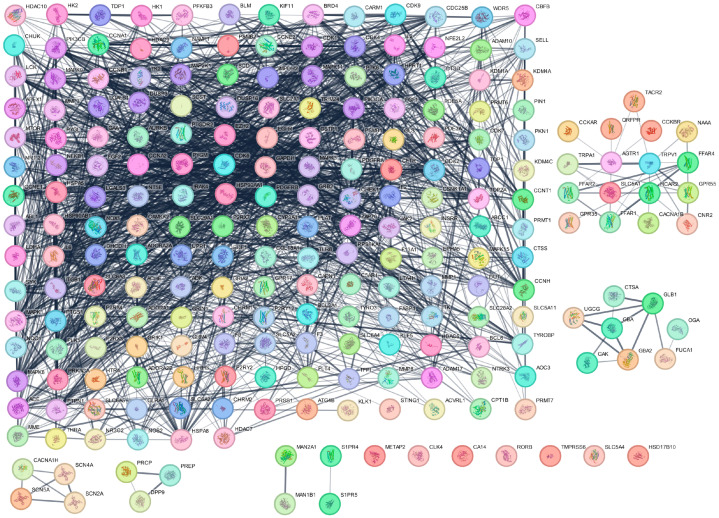
Protein–protein interaction (PPI) network of candidate targets. The diagram illustrates the interactions among proteins identified as potential targets of empagliflozin in CKD. Each node represents a protein, while edges indicate experimentally validated or predicted associations derived from multiple evidence sources. Node labels correspond to protein names, and edge colors reflect the confidence level of supporting evidence. To enhance readability, MCL clustering was applied to group related nodes, and a clearer grid-based layout was adopted. The resulting visualization highlights the dense connectivity and central roles of hub proteins, underscoring their importance in regulating cellular processes and their potential involvement in CKD-related pathways.

To explore the interactions among candidate proteins, a PPI network was generated using the STRING database, incorporating 221 shared targets. As illustrated in Figure 3, the network depicts the intricate interconnections between proteins, where each node represents a protein and each edge denotes experimentally validated or predicted interactions derived from curated databases, experimental evidence, and co-expression data. The thickness of the connecting lines reflects the strength of these interactions. The resulting network revealed a highly interconnected system, emphasizing the complexity of functional relationships and highlighting central hub proteins that may play critical roles in regulating cellular signaling and biological processes. To improve clarity, the PPI network was simplified by applying MCL clustering to group related nodes. This analysis revealed one main network containing the majority of highly interconnected hub proteins, along with five smaller sub-networks representing functionally related modules. A clear grid-based layout was adopted to enhance readability and highlight the key proteins. During this process in Cytoscape, several proteins, including SLC5A4, CLK4, CA14, METAP2, TMPRSS6, RORB, HSD17B10, MAN1B1, and MAN2A1, were excluded due to their lack of connectivity with the main network. The primary PPI network was subsequently subjected to further topological analysis in Cytoscape to identify hub proteins and characterize network properties. Overall, these findings indicate that the shared targets are embedded within essential molecular pathways rather than functioning independently, offering mechanistic insights into how empagliflozin may exert therapeutic effects in CKD through multi-target and pathway-level modulation.

### 3.5. Top 10 Targets Identification by cytoHubba

**Figure 4:**
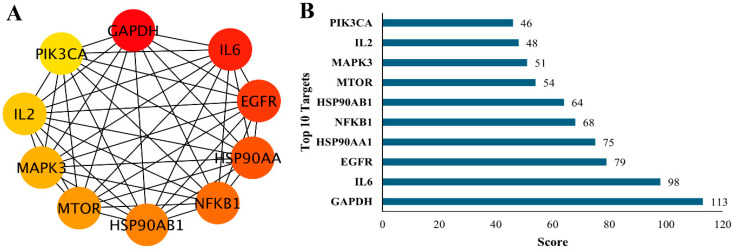
Network and ranking analysis of candidate targets. (**A**) Protein–protein interaction (PPI) network of 10 key targets associated with the study, where nodes represent individual proteins and edges indicate predicted or known interactions. Node color intensity (yellow to red) reflects relative importance or connectivity within the network. (**B**) Bar chart ranking the top 10 targets based on their network scores, with GAPDH, IL6, EGFR, HSP90AA1, and NFKB1 emerging as the highest-scoring nodes, followed by HSP90AB1, MTOR, MAPK3, IL2, and PIK3CA. Together, these analyses highlight central hub proteins that may play critical roles in the biological processes under investigation and represent potential therapeutic or mechanistic targets.

To pinpoint the most influential regulatory proteins within the empagliflozin–CKD network, the cytoHubba plugin in Cytoscape 3.10.1 was applied to rank hub genes according to their degree of connectivity. This analysis identified ten highly interconnected proteins that varied in their relative importance within the PPI network (Figure 4A). In the visualization, node color intensity corresponded to centrality, with deeper shades indicating stronger connectivity. The ranking results highlighted GAPDH, IL6, EGFR, HSP90AA1, and NFKB1 as the top-scoring hub proteins, followed by HSP90AB1, MTOR, MAPK3, IL2, and PIK3CA (Figure 4B). The prominence of these hub proteins suggests that they serve as critical regulators of signaling pathways and CKD pathophysiology. These hub proteins represent highly connected nodes that are not only central in network topology but also biologically relevant to CKD pathogenesis. In CKD, top 10 hub proteins encompass metabolic dysregulation (GAPDH, MTOR, PIK3CA), inflammatory signaling (IL6, IL2, NFKB1), stress responses (HSP90AA1 and HSP90AB1), and maladaptive pathways and tubular epithelial cell proliferation and repair (EGFR, MAPK3). Together, these proteins integrate diverse pathogenic mechanisms that drive CKD progression. Their identification highlights potential therapeutic targets and provides mechanistic insight into disease biology. Collectively, these findings indicate that empagliflozin might exert its therapeutic effects by simultaneously modulating multiple central targets, thereby influencing interconnected biological processes rather than acting through a single molecular pathway.

### 3.6. Results of Gene Ontology (GO) Enrichment Analysis

**Figure 5:**
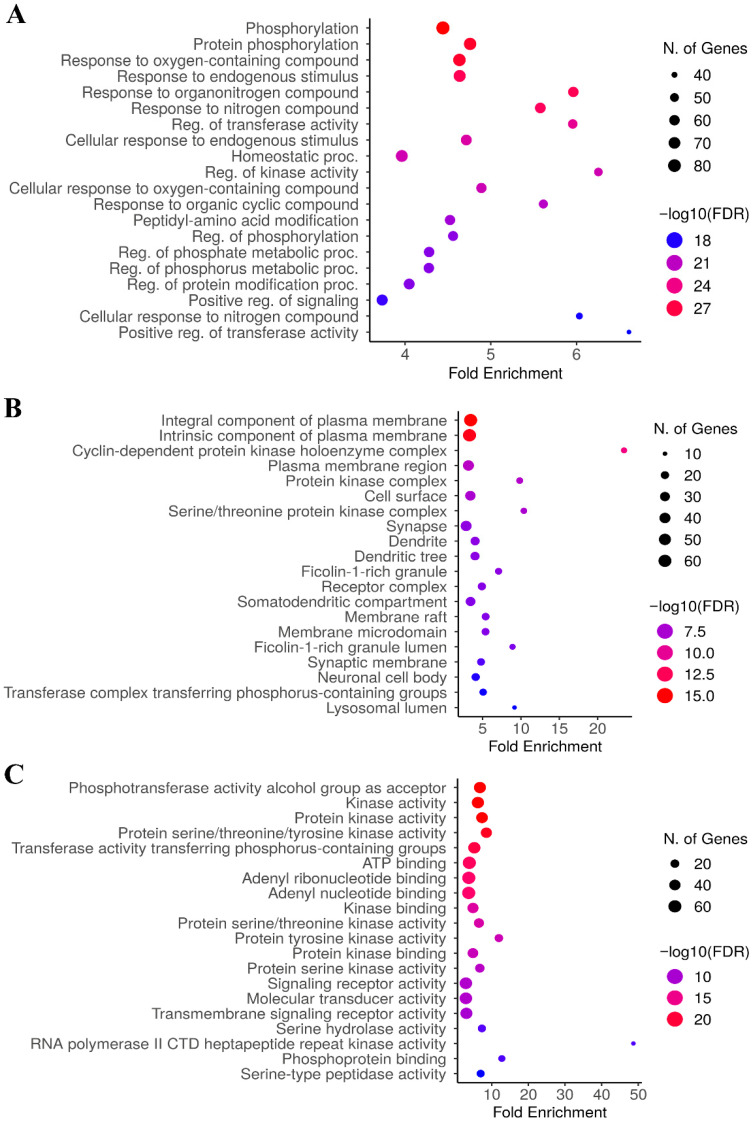
GO enrichment analysis of the 221 identified candidate targets. The figure displays a bar chart of the top 20 enriched GO terms, highlighting: (**A**) Biological Process (BP), (**B**) Cellular Component (CC), and (**C**) Molecular Function (MF) enrichment analysis for targets of empagliflozin (*p* value < 0.05). Collectively, these results indicate that the candidate targets are strongly associated with phosphorylation-related signaling, membrane-associated complexes, and kinase-mediated regulatory functions, underscoring their potential roles in cellular signaling and disease modulation.

GO enrichment analysis of the 221 shared targets between empagliflozin and CKD revealed significant enrichment across BP, CC, and MF categories (Table 3 and Figure 5), performed using ShinyGO version 0.85. In the BP domain, the most enriched terms were strongly associated with phosphorylation (GO:0016310), protein phosphorylation (GO:0006468), responses to oxygen-containing and nitrogen compounds (GO:1901700), response to endogenous stimulus (GO:0009719), and response to organonitrogen compound (GO:0010243), suggesting that shared targets are heavily involved in regulating signal transduction and adaptive stress responses relevant to CKD pathophysiology (Figure 5A). Within the CC category, enriched terms were predominantly linked to integral component of plasma membrane (GO:0005887), intrinsic component of plasma membrane (GO:0031226), cyclin-dependent protein kinase holoenzyme complex (GO:0000307), plasma membrane region (GO:0098590), and protein kinase complex (GO:1902911), highlighting the importance of membrane-anchored signaling machinery in mediating empagliflozin–CKD interactions (Figure 5B). For MF, the top enriched terms included phosphotransferase activity, alcohol group as acceptor (GO:0016773), kinase activity (GO:0016301), protein kinase activity (GO:0004672), protein serine/threonine/tyrosine kinase activity (GO:0004712), and transferase activity transferring phosphorus-containing groups (GO:0016772), reflecting the importance of phosphorylation and signal transduction processes in the shared empagliflozin–CKD targets (Figure 5C). Collectively, GO enrichment analysis of the 221 shared empagliflozin–CKD targets demonstrated significant involvement in phosphorylation-related biological processes, membrane-associated signaling complexes, and kinase-driven molecular functions, underscoring their central roles in signal transduction and disease-relevant pathways.

### 3.7. Results of KEGG Enrichment Analysis

**Figure 6:**
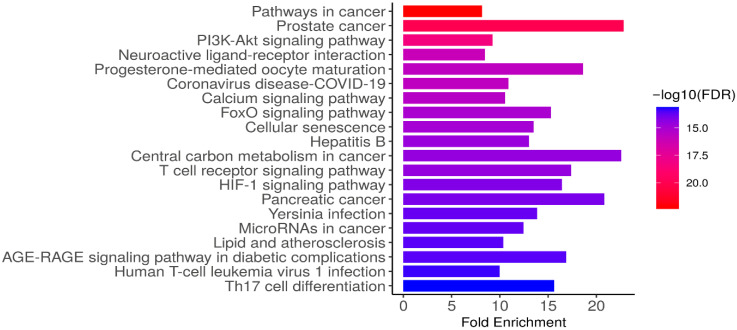
KEGG pathway enrichment analysis of the 221 identified candidate targets. The bar chart illustrates the significantly enriched KEGG pathways associated with the predicted targets. The *x*-axis represents the fold enrichment of each pathway, while the color gradient of the bars corresponds to the statistical significance, expressed as −log10(FDR), with deeper red shades indicating higher significance (enrichment profiles retrieved from KEGG database: https://www.kegg.jp/kegg/kegg1.html; accessed on 6 February 2026).

KEGG pathway enrichment analysis of the 221 shared targets between empagliflozin and CKD revealed significant clustering in pathways related to cancer, immune regulation, metabolic signaling, and inflammatory responses (Table 4 and Figure 6), performed using ShinyGO version 0.85. The most enriched pathways included pathways in cancer (hsa05200, 41 genes, FDR = 4.12 × 10^−23^) and prostate cancer (hsa05215, 21 genes, FDR = 8.87 × 10^−21^), reflecting the strong involvement of oncogenic signaling networks. Importantly, several canonical signaling cascades were highlighted, such as the PI3K-Akt pathway (hsa04151, 31 genes, FDR = 4.73 × 10^−19^), FoxO signaling (hsa04068, 19 genes, FDR = 7.77 × 10^−16^), HIF-1 signaling (hsa04066, 17 genes, FDR = 6.83 × 10^−15^), and calcium signaling (hsa04020, 24 genes, FDR = 3.30 × 10^−16^), all of which are central to cell survival, stress responses, and metabolic regulation (Figure 6). Pathways associated with immune and inflammatory processes were also enriched, including T cell receptor signaling (hsa04660, 17 genes), Th17 cell differentiation (hsa04659, 16 genes), and the AGE-RAGE signaling pathway in diabetic complications (hsa04933, 16 genes), underscoring their relevance to CKD pathophysiology. Additional enrichment was observed in pathways related to cellular senescence, lipid metabolism and atherosclerosis, neuroactive ligand-receptor interaction, and viral infections (COVID-19, hepatitis B, HTLV-1), suggesting broad involvement of shared targets in stress, immune, and metabolic networks. As shown in Table 4, several enriched KEGG pathways include broad categories such as cancer-related pathways. Although these may appear less directly relevant to CKD, they often represent shared molecular mechanisms, such as PI3K-Akt signaling, HIF-1 signaling, AGE-RAGE signaling in diabetic complications, and T cell receptor signaling, that are central to CKD progression through processes including inflammation, fibrosis, oxidative stress, and tubular injury. Collectively, these findings indicate that empagliflozin might exert therapeutic effects in CKD by modulating interconnected pathways that regulate inflammation, oxidative stress, vascular dysfunction, and metabolic homeostasis, thereby providing mechanistic insights into its multi-target mode of action.

### 3.8. Molecular Docking Results

Molecular docking analysis was conducted to assess the binding affinity of empagliflozin toward proteins implicated in CKD pathogenesis (Table 5). Empagliflozin demonstrated favorable binding energies across multiple targets, in several cases comparable to or stronger than those of the reference co-crystallized inhibitors. The docking scores of empagliflozin were directly compared with those of the co-crystallized ligands, further supporting the validity of the docking protocol. Notably, empagliflozin exhibited strong binding to HSP90AB1 with binding energy at −10.85 kcal/mol and inhibition constant (Ki) at 11.09 nM, MAPK3 with binding energy at −9.46 kcal/mol (Ki = 116.72 nM), EGFR with binding energy at −9.38 kcal/mol (Ki = 133.37 nM), and mTOR with binding energy at −8.89 kcal/mol (Ki = 304.24 nM), indicating high affinity for signaling proteins central to CKD progression. Against IL6 (−7.65 kcal/mol) and PIK3CA (−7.73 kcal/mol), empagliflozin exhibited micromolar inhibition constants, closely matching or even surpassing their respective positive controls. Likewise, docking with NFKB1 as binding energy at −7.32 kcal/mol (Ki = 4.33 µM) and IL2 as binding energy at −7.46 kcal/mol (Ki = 3.42 µM) indicated potential binding affinities with key immune-regulatory proteins. These findings suggest that empagliflozin may engage multiple hub proteins involved in inflammation, metabolism, and signaling pathways. While docking results are predictive and require experimental validation, they provide preliminary support for a potential multi-target mechanism of action in CKD.

**Table 5:** In silico molecular docking of empagliflozin against proteins implicated in CKD pathogenesis.

No	Protein Name	PDB	Compound and Positive Control	Binding Energies (kcal/mol)	Inhibition Constant
1	GAPDH	6M61	Empagliflozin	−6.61	14.3 µM
			heptelidic acid	−6.64	13.6 µM
2	IL6	1ALU	Empagliflozin	−7.65	2.47 µM
			DB08402	−7.54	2.95 µM
3	EGFR	8A27	Empagliflozin	−9.38	133.37 nM
			KY91102	−12.91	343.44 pM
4	HSP90AA1	5h22	Empagliflozin	−8.85	326.76 nM
			7FT301	−9.60	92.63 nM
5	NFKB1	1SVC	Empagliflozin	−7.32	4.33 µM
			Parthenolide	−6.99	7.58 µM
6	HSP90AB1	6N8Y	Empagliflozin	−10.85	11.09 nM
			KFY301	−7.27	4.71 µM
7	MTOR	3TL5	Empagliflozin	−8.89	304.24 nM
			GDC-0980	−9.95	51.18 nM
8	MAPK3	4QTB	Empagliflozin	−9.46	116.72 nM
			SCH772984	−13.42	144.92 pM
9	IL2	1PW6	Empagliflozin	−7.46	3.42 µM
			FRB201	−10.33	26.62 nM
10	PIK3CA	5XGH	Empagliflozin	−7.73	2.16 µM
			84U1101	−9.20	181.21 nM

### 3.9. Binding Interactions of Empagliflozin and KFY301 with HSP90AB1

**Figure 7:**
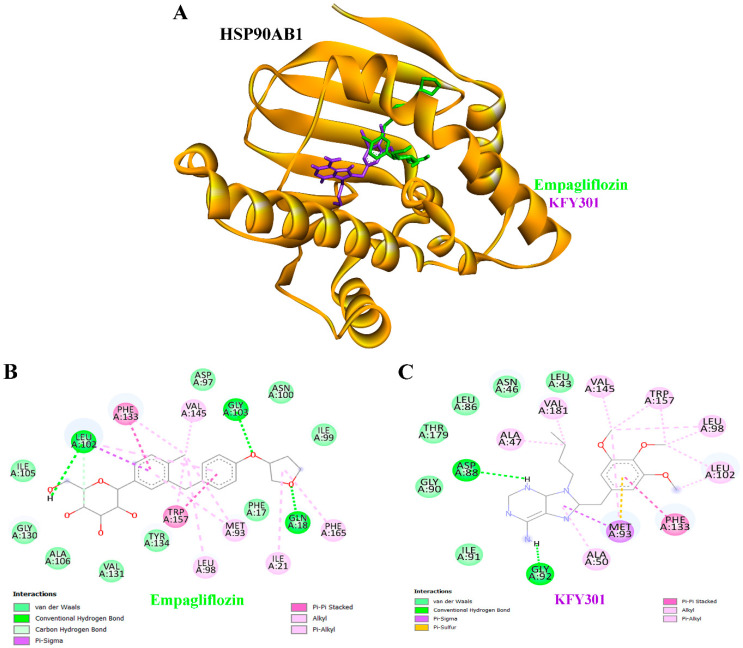
Binding interactions of empagliflozin and KFY301 with HSP90AB1. (**A**) Three-dimensional ribbon structure of HSP90AB1 (orange) showing the binding orientation of empagliflozin (green) and KFY301 (purple) within the active site. (**B**) Two-dimensional interaction diagram of empagliflozin with HSP90AB1, illustrating hydrogen bonds, alkyl contacts, and π-stacking interactions with key residues. (**C**) Two-dimensional interaction diagram of KFY301 with HSP90AB1, highlighting hydrogen bonding, hydrophobic contacts, and π-stacking interactions that stabilize ligand binding. Comparative visualization of these panels underscores the similarities and differences in binding modes between the test compound and reference ligand.

Molecular docking analysis of HSP90AB1 indicated that both empagliflozin and the reference ligand KFY301 were accommodated within the protein’s active site, though with distinct interaction profiles (Figure 7A). Empagliflozin engaged residues such as GLN18, LEU102, and GLY103 through hydrogen bonding, along with aromatic contacts involving PHE133 and TRP157, suggesting a stable binding orientation (Figure 7B). In comparison, KFY301 formed hydrogen bonds with ASP88 and GLY92 and established a broader network of hydrophobic and aromatic interactions, including contacts with ALA47, MET93, LEU98, and TRP157 (Figure 7C). Overall, the docking results suggest that while both ligands occupy the HSP90AB1 active site, their distinct interaction networks, empagliflozin favoring hydrogen and aromatic contacts, and KFY301 engaging a wider hydrophobic profile, may reflect alternative modes of protein modulation. These findings are predictive and should be perform experimental validation.

### 3.10. Binding Interactions of Empagliflozin and SCH772984 with MAPK3

**Figure 8:**
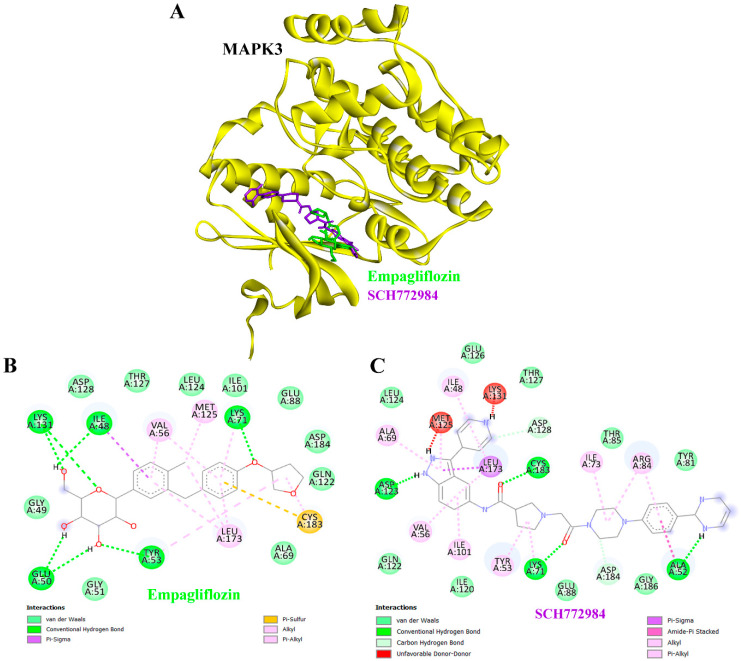
Binding interactions of empagliflozin and SCH772984 with MAPK3. (**A**) Three-dimensional structure of MAPK3 (yellow ribbon) showing the binding orientation of empagliflozin (green) and the reference inhibitor SCH772984 (magenta) within the active site. (**B**) Two-dimensional interaction diagram of empagliflozin with MAPK3, highlighting hydrogen bonds (green dashed lines), hydrophobic contacts (red arcs), and other key molecular interactions with surrounding residues. (**C**) Two-dimensional interaction diagram of SCH772984 with MAPK3, illustrating hydrogen bonding, hydrophobic interactions, and additional contacts that stabilize ligand binding. Together, these panels provide a comparative view of the binding modes and interaction profiles of the test compound and reference inhibitor.

Molecular docking analysis of MAPK3 suggested that both empagliflozin and the reference inhibitor SCH772984 could be accommodated within the active site pocket, though with distinct orientations and interaction profiles (Figure 8A). Empagliflozin engaged residues such as ILE48, GLU50, TYR53, LYS71, and LYS131 through hydrogen bonding, along with hydrophobic and other non-covalent contacts that supported a plausible binding conformation (Figure 8B). SCH772984, in contrast, formed hydrogen bonds with ALA52, LYS71, ASP123, ASP128, and CYS183, together with a broader set of hydrophobic interactions that reinforced its affinity for MAPK3 (Figure 8C). Overall, the docking results might indicate that both ligands occupy the MAPK3 active site but interact through distinct networks, highlighting potential differences in their binding modes.

### 3.11. Comparative Molecular Docking Analysis of Empagliflozin and KY91102 with EGFR

**Figure 9:**
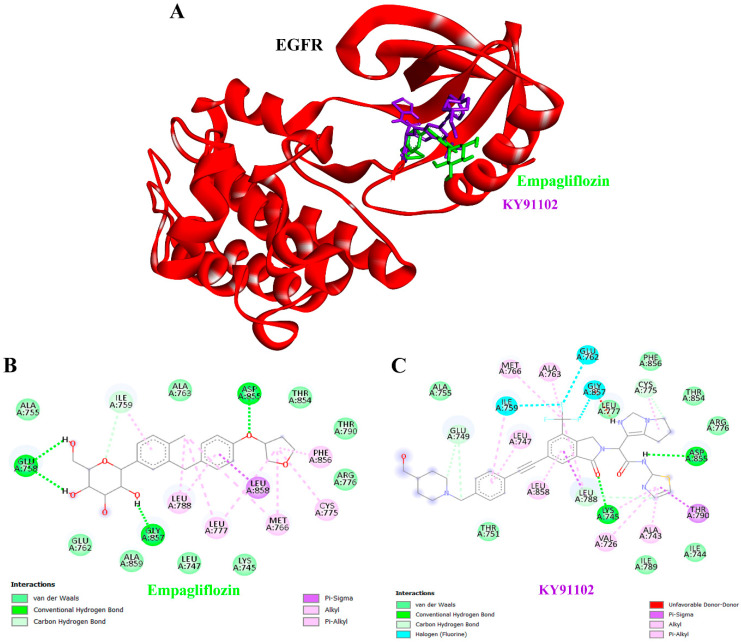
Binding interactions of empagliflozin and KY91102 with EGFR. (**A**) Three-dimensional ribbon structure of EGFR (red) showing the binding orientation of empagliflozin (green) and KY91102 (purple) within the active site pocket. (**B**) Two-dimensional interaction diagram of empagliflozin with EGFR, highlighting van der Waals forces, conventional hydrogen bonds, carbon–hydrogen bonds, and π–sigma interactions with key residues. (**C**) Two-dimensional interaction diagram of KY91102 with EGFR, illustrating van der Waals contacts, hydrogen bonding, carbon–hydrogen bonds, π–sigma, π–π stacking, and π–alkyl interactions with surrounding residues. Comparative visualization of these panels emphasizes the distinct binding modes and stabilizing interactions of the test compound versus the reference ligand.

The molecular docking visualization of EGFR revealed distinct binding interactions for empagliflozin compared with the reference ligand KY91102 (Figure 9A). In the three-dimensional structure, both compounds were positioned within the active site pocket of EGFR, but their orientations and interaction profiles differed. Empagliflozin established stabilizing contacts through conventional hydrogen bonds and carbon–hydrogen bonds with key residues such as GLU758, ILE759, ASP855, and GLY857, indicating a favorable binding conformation. Moreover, empagliflozin formed van der Waals contacts, π–sigma interactions, and π–alkyl interactions within the EGFR active site (Figure 9B). KY91102 exhibited a broader spectrum of interactions, including hydrogen bonds with GLU749, LYS745, LEU788, CYS775, and ASP855, along with van der Waals contacts, π–sigma, halogen, and π–alkyl interactions (Figure 9C). Therefore, docking visualization showed that both empagliflozin and KY91102 occupy the EGFR active site pocket with key amino acids.

### 3.12. Molecular Dynamic Simulation Analysis

**Figure 10:**
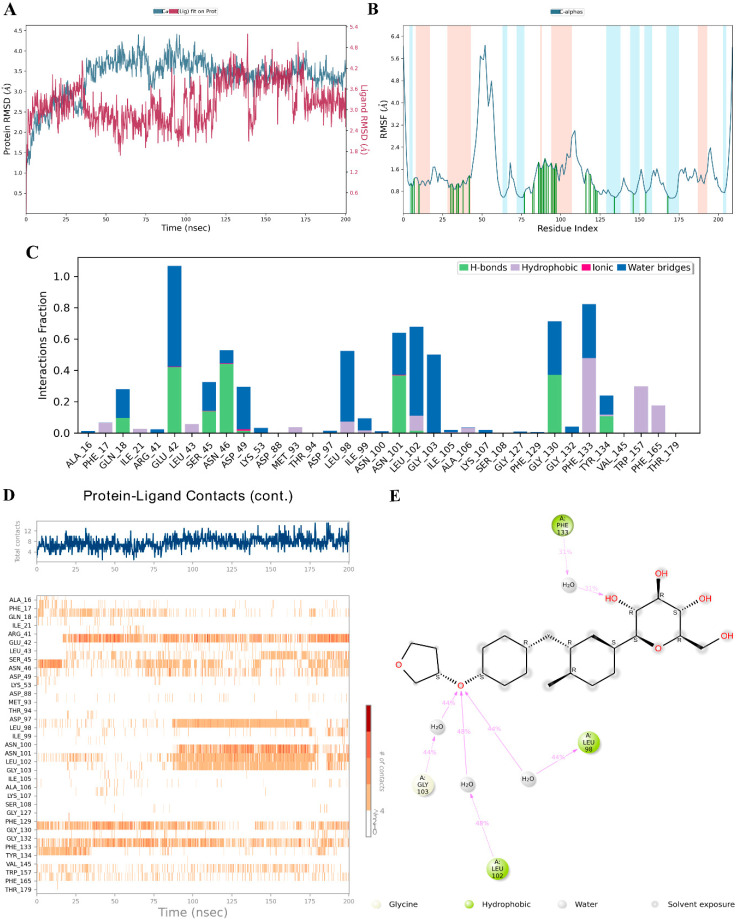
Molecular dynamics (MD) simulation of the empagliflozin–HSP90AB1 complex over a 200-ns trajectory. (**A**) RMSD profiles showing protein–ligand stability. (**B**) RMSF plots highlighting residue flexibility. (**C**) Timeline interaction analysis across the simulation. (**D**) Post-simulation contact maps depicting amino acid interactions. (**E**) 2D interaction diagram of empagliflozin with HSP90AB1.“#” in the figure indicates the number or amount of contacts.

Empagliflozin emerged as the most promising candidate due to its strong binding affinity with key HSP90AB1 residues and high docking score, with its interaction profile further validated through 200 ns molecular dynamics simulations. The simulations revealed stable binding and favorable interaction dynamics with the target protein. The protein RMSD remained within 3.0–4.2 Å during the 50–200 ns timeframe, indicating structural stability (Figure 10A). RMSF analysis showed moderate fluctuations in residues 40–60, particularly in loop regions, while structured domains remained stable, reflecting localized flexibility without compromising global integrity (Figure 10B). Empagliflozin maintained stable interactions through hydrogen bonds with GLN18, GLU42, SER45, ASN46, ASN101, GLY130, and TYR134, complemented by hydrophobic contacts, ionic interactions, and water-mediated bridges (Figure 10C). These interactions persisted throughout the simulation, as shown by the contact frequency heatmap, confirming the ligand’s consistent engagement with the protein (Figure 10D). The 2D interaction diagram further highlighted hydrogen bonding, hydrophobic contacts, and water-mediated interactions, supporting a well-anchored binding conformation (Figure 10E). Overall, the simulation indicates that empagliflozin maintains a stable and multi-faceted interaction with the protein target, suggesting potential pharmacological relevance through sustained molecular engagement.

## 4. Discussion

Empagliflozin demonstrates strong oral drug-likeness, with physicochemical and pharmacokinetic properties largely within optimal ranges; despite a slightly elevated TPSA, its permeability remains acceptable (Table 1). It complies with multiple drug-likeness filters, including Lipinski’s rule of five [34], and shows favorable pharmacokinetic and ADMET characteristics such as rapid absorption (Tmax 1.5–2 h, bioavailability ~60–70%), moderate distribution (~86% protein binding), minimal hepatic metabolism via glucuronidation, and a terminal half-life of ~12 h, supporting once-daily dosing [35]. Empagliflozin exhibits limited CYP inhibition, low risk of drug–drug interactions, and a generally favorable safety profile aside from a flagged hERG II interaction. Clinical evidence, including meta-analyses of randomized trials, confirms that SGLT2 inhibitors such as empagliflozin reduce cardiovascular hospitalizations and kidney injury in patients with HFpEF, without significantly affecting cardiovascular or all-cause mortality [36].

CKD is a progressive disorder characterized by declining kidney function, inflammation, and metabolic abnormalities that can lead to ESRD and cardiovascular complications, while DKD, a major subtype driven by diabetes, accounts for most CKD cases through fibrosis and structural damage [37,38]. Empagliflozin provides renal and cardiovascular protection by improving glycemic control and mitigating disease progression through mechanisms beyond glucose lowering [39]. Its reno-protective effects include reducing intraglomerular pressure, lowering hyperfiltration, improving tubular oxygenation, and modulating inflammatory and fibrotic pathways, as supported by clinical and mechanistic studies [40]. Empagliflozin benefits CKD by going beyond its initial hemodynamic effects. It also impacts molecular pathways such as PI3K/Akt, HIF-1, and AGE-RAGE signaling, which are involved in the development of kidney disease [41]. By influencing these pathways, empagliflozin helps reduce oxidative stress and cellular damage, leading to potential benefits for the kidneys, as shown in studies on both diabetic and autoimmune kidney diseases [42]. These pleiotropic actions highlight empagliflozin’s potential to slow CKD progression and reduce associated complications, supporting its role as a multifaceted therapeutic option in the management of kidney disease. Empagliflozin’s reno-protective actions are further supported by GO molecular function and KEGG pathway enrichment analyses, which reveal that its shared CKD targets are strongly associated with phosphorylation and signal transduction processes, including kinase and protein kinase activities. Key signaling cascades such as PI3K/Akt, FoxO, HIF-1, and calcium signaling were highlighted, underscoring their central roles in cell survival, stress adaptation, and metabolic regulation. In addition, enrichment of immune and inflammatory pathways, including T cell receptor signaling, Th17 cell differentiation, and the AGE-RAGE axis, aligns with evidence that empagliflozin modulates inflammatory and fibrotic processes beyond its hemodynamic effects. Although several enriched KEGG pathways are broadly categorized as cancer-related, these annotations reflect shared molecular mechanisms rather than direct oncogenic processes. Importantly, pathways such as PI3K-Akt, HIF-1, AGE-RAGE, and T cell receptor signaling are highly pertinent to CKD management, as they contribute to inflammation, fibrosis, oxidative stress, and tubular injury [41]. By emphasizing these CKD-related mechanisms, our analysis highlights the biological relevance of empagliflozin’s predicted targets, while acknowledging that broader cancer-related pathways represent overlapping signaling networks common to chronic disease progression. Taken together, these findings reinforce clinical and mechanistic evidence that empagliflozin not only lowers intraglomerular pressure and reduces hyperfiltration but also influences key molecular networks driving CKD progression, thereby offering a multifaceted therapeutic benefit.

Network pharmacology analysis combined with target identification using CytoHubba ranked GAPDH, IL6, EGFR, HSP90AA1, and NFKB1 as the top hub proteins, followed by HSP90AB1, MTOR, MAPK3, IL2, and PIK3CA (Figure 4B). The prominence of these proteins underscores their central roles as regulators of signaling pathways and contributors to CKD pathophysiology. By integrating our computational findings with established biological evidence, we highlight their translational relevance and strengthen the clinical significance of this work. Network pharmacology identified GAPDH as a key hub protein in CKD. Although traditionally used as a housekeeping gene, GAPDH shows variable expression in diseased kidneys, raising concerns about its reliability as a control [43]. Beyond glycolysis, it contributes to oxidative stress, apoptosis, and inflammatory signaling, processes central to CKD progression, suggesting its potential as a marker of metabolic stress and disease progression, thereby reinforcing the translational relevance of our findings [43]. IL-6 is strongly implicated in CKD, where its persistent elevation promotes inflammation, renal injury, fibrosis, and progressive loss of kidney function [44]. Elevated IL-6 levels are associated with faster CKD progression, dialysis dependence, higher mortality, and increased cardiovascular risk, underscoring its role in both renal and systemic complications [45]. Given its correlation with disease severity and outcomes, IL-6 is recognized not only as a prognostic biomarker but also as a potential therapeutic target, highlighting the translational relevance of our findings [44,45]. EGFR, expressed in renal tubular epithelial cells, is activated during kidney injury to support survival and repair, but sustained activation promotes fibrosis, inflammation, and extracellular matrix accumulation, driving CKD progression [46]. Experimental studies show that EGFR inhibition reduces renal fibrosis and slows disease advancement, while clinical observations link elevated EGFR activity to worsening renal function and structural damage [47]. These findings highlight EGFR’s dual role as both a potential therapeutic target and a biomarker in CKD [46,47]. HSP90, a cytosolic heat shock protein that stabilizes kinases and transcription factors, is frequently upregulated in CKD under persistent oxidative stress and inflammation [48]. By supporting proteins involved in TGF-β, NF-κB, and PI3K-Akt signaling, it contributes to renal fibrosis and disease progression [49]. Clinical evidence links elevated HSP90, particularly HSP90AA1 and HSP90AB1, with the severity of kidney injury, positioning them as stress biomarkers and potential therapeutic targets in CKD [50], thereby reinforcing the translational relevance of our findings. NFKB1, encoding the p50 subunit of NF-κB, is a central regulator of inflammation and immune responses. Its persistent activation in CKD promotes cytokine release, including IL-6, TNF-α, oxidative stress, and fibrotic signaling, thereby accelerating renal injury and disease progression [51]. Elevated NF-κB activity is linked to disease severity, poor outcomes, and complications such as hypertension, underscoring its crucial role in CKD pathophysiology and highlighting its translational as both a biomarker and therapeutic target [52]. mTOR is a central regulator of cell growth, metabolism, and stress responses in the kidney, but chronic activation of mTORC1 promotes glomerular hypertrophy, podocyte injury, and fibrosis, driving CKD progression [53]. Normally balanced by AMPK, disruption of this axis contributes to diabetic nephropathy and other CKD forms [54]. Experimental studies show that mTOR inhibitors can reduce fibrosis, inflammation, and proteinuria, though clinical use is complicated by risks such as proteinuria and podocyte dysfunction, requiring careful monitoring and dose adjustment [55]. Therefore, mTOR plays a dual role in CKD pathophysiology, underscoring its translational relevance as both a therapeutic target and a biomarker. MAPK3, a key component of the ERK1/2 branch of the MAPK pathway, regulates cell growth and survival but becomes pathogenic in CKD when persistently activated by stress signals, cytokines, or hyperglycemia [56]. Overactivation of MAPK3 pathway drives fibrosis, mesangial proliferation, podocyte injury, and proteinuria, particularly in diabetic nephropathy. Experimental inhibition of MAPK/ERK signaling reduces renal inflammation and fibrosis, while elevated MAPK activity in patient biopsies correlates with CKD severity and progression [57]. IL-2, a key cytokine for T-cell proliferation and survival, is dysregulated in CKD, where reduced activity impairs regulatory T-cell function, promotes inflammation, and accelerates renal injury and fibrosis [58]. Lower IL-2 levels and signaling in CKD patients correlate with immune dysfunction and higher infection risk [59]. While low-dose IL-2 therapy shows promise in restoring immune balance and reducing inflammation, its role requires further study; importantly, IL-2 pathways remain central targets of immunosuppressive drugs in transplantation [60]. PIK3CA, encoding the PI3Kα catalytic subunit, is central to the PI3K/Akt pathway regulating cell growth and survival. In CKD, its dysregulation promotes mesangial proliferation, extracellular matrix accumulation, fibrosis, and podocyte injury [61]. Experimental inhibition of PI3Kα reduces renal inflammation and fibrosis in glomerulonephritis models, while elevated PI3K/Akt signaling in patients with diabetic nephropathy and other CKD forms correlates with disease severity and progression [62]. The top ten targets identified through network pharmacology analysis are strongly implicated in CKD pathophysiology. Most of these hub proteins demonstrate translational relevance as both biomarkers and therapeutic targets, given their central roles in inflammation, fibrosis, and metabolic regulation. Collectively, they represent promising candidates that may mediate or enhance the reno-protective effects of empagliflozin.

Docking results highlight empagliflozin’s polypharmacological potential in CKD. Unlike highly selective inhibitors, it shows broad affinity for multiple hub proteins, including kinases, growth factor receptors, and immune regulators—reflecting the complex pathophysiology of CKD, where inflammation, metabolic dysregulation, and aberrant signaling converge. Notably, its strong binding to HSP90AB1 is of particular significance (Table 5, Figure 7). Strong binding to HSP90AB1 is particularly significant, as this constitutively expressed chaperone stabilizes diverse client proteins, including kinases, transcription factors, and signaling molecules, that sustain pathological pathways in CKD and drive disease progression [63]. By supporting proteins in the AKT, MAPK, and mTOR pathways, HSP90AB1 promotes survival and stress responses [64]. Its interaction with mitochondrial proteins leads to excessive ROS production, inducing oxidative stress, tubular cell damage, and nephron loss, thereby accelerating CKD progression [65]. HSP90AB1 enhances renal inflammation by activating the NF-κB signaling pathway, which is consistently upregulated in both human kidney disease and animal models of renal injury [66]. Through this pathway, NF-κB drives inflammatory responses across multiple cell types, including renal parenchymal cells, innate immune cells, and lymphocytes, thereby contributing to the progression of kidney damage [66]. Taken together, these observations suggest that HSP90AB1 could act as a hub regulator in CKD progression, and its modulation represents a hypothesis that warrants experimental validation as a potential therapeutic strategy. KFY301 and PU-11-trans were included in this study because they are co-crystallized ligands of HSP90AB1 confirmed by X-ray diffraction. PU-11-trans binds within the ATP-binding pocket of HSP90AB1, inserting its purine ring into the central cavity and positioning its tri-methoxy phenyl group in the conserved hydrophobic site formed by MET98, LEU103, LEU107, ALA111, PHE138, TYR139, VAL150, and TRP162, while interacting with surrounding residues, including PHE22, ALA47, ASN51, ALA55, ASP93, GLY97, GLY135, VAL136, THR184, and VAL186 [67]. Molecular docking in this study showed that both empagliflozin and KFY301 can bind to the HSP90AB1 active site, though with distinct interaction patterns (Figure 7A). Empagliflozin’s unique hydrogen bonds and π-interactions suggest a different binding orientation, which may result in downstream effects beyond glucose lowering. HSP90AB1 inhibitors have been proposed as therapeutic agents in CKD by targeting key processes such as renal fibrosis and cellular senescence. They may reduce fibrosis by promoting degradation of the transforming growth factor-β type II receptor (TβRII), thereby limiting scar tissue formation in the kidney. In addition, these inhibitors may alleviate cellular senescence in renal cells by enhancing autophagy, offering a potential strategy for treating both acute and chronic kidney injury [68]. Empagliflozin protects the kidney through multiple mechanisms beyond glucose lowering. By inhibiting SGLT2 in the proximal tubule, it reduces glucose and sodium reabsorption, restores tubule-glomerular feedback, and lowers intraglomerular pressure, while also improving blood pressure, vascular stiffness, and metabolic efficiency. In addition, it exerts anti-inflammatory and anti-fibrotic effects by reducing oxidative stress, inflammation, and fibrosis, collectively slowing CKD progression [69]. Previous studies have shown that empagliflozin downregulates both HSP90 and TGF-β expression, suggesting that its cardioprotective effects in patients with heart failure with preserved ejection fraction (HFpEF) extend beyond hemodynamic and metabolic regulation to include direct modulation of pro-fibrotic and stress-related pathways [70]. Empagliflozin modulates HSP90 expression and activity, contributing to its cardioprotective effects by regulating stress-related and inflammatory pathways. By reducing HSP90 levels, it disrupts pro-inflammatory and pro-fibrotic signaling complexes, thereby diminishing oxidative stress, fibrosis, and inflammation. This downregulation also limits activation of key pathways such as NF-κB and TGF-β, which are central to renal and cardiac injury [71]. Molecular dynamics simulations further validated empagliflozin’s binding, with RMSD values of 3.0–4.2 Å confirming protein stability and consistent ligand orientation (Figure 10A). RMSF analysis indicated localized flexibility without loss of global integrity. Empagliflozin maintained stable hydrogen bonds with GLN18, GLU42, SER45, ASN46, ASN101, GLY130, and TYR134, supported by hydrophobic, ionic, and water-mediated interactions throughout the 200-ns trajectory (Figure 10C,D). Together, these findings suggest that empagliflozin achieves strong affinity and sustained engagement with HSP90AB1, highlighting its might potential as a candidate for modulation of this target. Our proposed experiments, such as modulating HSP90AB1 in renal tubular cells under hyperglycemic conditions and assessing downstream NF-κB and fibrotic markers, offer a clear framework for validating and extending our computational predictions.

Moreover, docking analysis showed that empagliflozin binds favorably to the MAPK3 active site through hydrogen bonds (ILE48, GLU50, TYR53, LYS71, and LYS131) and hydrophobic interactions, suggesting it may dampen pro-inflammatory and pro-fibrotic signaling. In contrast, the reference inhibitor SCH772984 formed a broader hydrogen-bonding network with extensive hydrophobic contacts, consistent with its strong inhibitory activity (Figure 8). MAPK3, also known as ERK1, is a central kinase in the MAPK/ERK signaling cascade, regulating cell proliferation, inflammation, and fibrosis—processes critically involved in CKD progression [72]. Aberrant MAPK3 activation has been linked to renal tubular injury, mesangial cell proliferation, and extracellular matrix accumulation, making it an attractive therapeutic target [73]. The MAPK3/ERK1 signaling pathway plays a multifaceted role in CKD progression, contributing to both tissue injury and maladaptive repair. Its activation has been associated with specific CKD subtypes, including IgA nephropathy [72]. While essential during kidney development, excessive activation in mature kidneys can trigger harmful responses such as increased profibrotic factor expression, uncontrolled cell proliferation, and disrupted autophagy, all of which drive disease advancement. Consequently, modulating this pathway represents a promising therapeutic approach for selected CKD conditions [74], and inhibition of MAPK/ERK signaling may help slow disease progression. Collectively, our findings suggest that empagliflozin’s interaction with MAPK3 could contribute to its reno-protective activity. By dampening MAPK3-mediated signaling, empagliflozin may reduce renal inflammation and fibrotic remodeling, supporting the concept of a multi-target therapeutic mechanism in CKD management. However, the limitations of our work are that the study is entirely computational, with network pharmacology offering only predictive insights rather than direct biological evidence. Therefore, further experimental validation is required to confirm the interactions between the identified hub targets and empagliflozin in the context of CKD management.

Molecular docking analysis of EGFR revealed that empagliflozin engages the receptor through a distinct interaction profile compared with the reference ligand KY91102 (Figure 9). Although both ligands occupy the same active site pocket, their orientations and stabilizing contacts differ, suggesting alternative mechanisms of receptor modulation. Empagliflozin binds stably to the EGFR pocket through hydrogen bonds with key residues (GLU758, ILE759, ASP855, GLY857), supported by van der Waals and π-type interactions (Figure 9B). The EGFR pathway plays a dual role, supporting normal kidney function while also contributing to CKD progression. Although EGFR activation is essential for kidney repair following acute injury, persistent and dysregulated signaling in CKD drives harmful processes such as inflammation, fibrosis, and epithelial–mesenchymal transition (EMT) [75]. EGFR can also be activated by molecules such as angiotensin II and TGF-β, forming a complex signaling network that promotes pathology [76]. Consequently, inhibition of the EGFR pathway is being explored as a therapeutic approach for CKD, though potential systemic side effects of certain inhibitors remain an important consideration. From a translational perspective, these results suggest that empagliflozin’s reno-protective effects may extend beyond direct interactions with CKD-related signaling proteins, including HSP90AB1, MAPK3, and EGFR. Nonetheless, because docking studies are predictive, experimental validation through enzymatic and cell-based assays is required to confirm these interactions and establish their biological significance. The EMPA-KIDNEY Collaborative Group (2024) reported that the EMPA-KIDNEY trial demonstrated empagliflozin significantly reduced the risk of kidney disease progression or cardiovascular death in patients with CKD, with progression occurring in 11.6% of those receiving empagliflozin compared to 15.2% on placebo [77]. The benefit was consistent across diverse primary kidney disease subgroups, including DKD, glomerular disease, hypertensive or renovascular disease, and other or unknown causes, with no evidence of heterogeneity. Importantly, empagliflozin slowed the chronic decline in kidney function, reducing the rate of eGFR loss by about 50%, and this effect was similar across all disease categories. Overall, these findings support empagliflozin as a broadly effective therapy to slow CKD progression and reduce the risk of kidney failure, regardless of underlying etiology [77]. Therefore, our systematic study of the mechanisms of empagliflozin in CKD, along with target identification, highlights how this drug modulates disease progression and demonstrates favorable outcomes in CKD patients, thereby reinforcing its clinical utility.

## 5. Conclusions

In conclusion, this study employed an integrated approach combining network pharmacology and molecular docking to elucidate the multi-target mechanisms underlying empagliflozin’s therapeutic potential in CKD. Initial analyses confirmed empagliflozin’s favorable physicochemical and ADMET properties, supporting its suitability as an orally active therapeutic agent. Network analysis identified 221 shared molecular targets between the drug and CKD-associated genes, with the top ten hub proteins being GAPDH, IL6, EGFR, HSP90AA1, NFKB1, HSP90AB1, MTOR, MAPK3, IL2, and PIK3CA. Enrichment analysis further revealed that these shared targets are critically involved in phosphorylation, signal transduction, and key signaling cascades central to CKD pathogenesis, including the PI3K-Akt, FoxO, HIF-1, and AGE-RAGE pathways. Molecular docking demonstrated empagliflozin’s polypharmacological potential through favorable binding energies across multiple targets, with particularly strong affinity for HSP90AB1, MAPK3, and EGFR. Collectively, these findings suggest potential pathways through which empagliflozin may influence inflammation, oxidative stress, and metabolic regulation in CKD, while underscoring the need for further in vitro validation.

## Figures and Tables

**Table 1:** In silico evaluation of empagliflozin’s physicochemical and drug-likeness properties using SwissADME tools.

Physicochemical Properties	
Formula	C_23_H_27_ClO_7_
Molecular weight	450.91
Num. heavy atoms	31
Num. arom. heavy atoms	12
Fraction Csp3	0.48
Num. rotatable bonds	6
Num. H-bond acceptors	7
Num. H-bond donors	4
Molar Refractivity	113.41
TPSA	108.61
**Lipophilicity**	
Log Po/w (iLOGP)	3.18
Log Po/w (XLOGP3)	2.03
Log Po/w (WLOGP)	1.29
Log Po/w (MLOGP)	0.70
Log Po/w (SILICOS-IT)	2.66
Consensus Log Po/w	1.97
**Water Solubility**	
Log S (ESOL)	−3.80
Solubility	7.06 × 10^−2^ mg/mL
Class	Soluble
Log S (Ali)	−3.94
Solubility	5.19 × 10^−2^ mg/mL
Class	Soluble
Log S (SILICOS-IT)	−4.25
Solubility	2.54 × 10^−2^ mg/mL
Class	Moderate soluble
**Drug-likeness**	
Lipinski	Yes
Ghose	Yes
Veber	Yes
Egan	Yes
Muegge	Yes
Bioavailability Score	0.55

**Table 2:** In silico estimation of empagliflozin’s pharmacokinetic and ADMET characteristics via pkCSM.

Property	Model Name	Predicted Value	Unit
**Absorption**	Water solubility	−3.56	Numeric (log mol/L)
	Caco2 permeability	−0.019	Numeric (log Papp in 10^−6^ cm/s)
	Intestinal absorption (human)	55.879	Numeric (% Absorbed)
	Skin Permeability	−2.744	Numeric (log Kp)
	P-glycoprotein substrate	Yes	Categorical (Yes/No)
	P-glycoprotein I inhibitor	Yes	Categorical (Yes/No)
	P-glycoprotein II inhibitor	Yes	Categorical (Yes/No)
**Distribution**	VDss (human)	−0.387	Numeric (log L/kg)
	Fraction unbound (human)	0.073	Numeric (Fu)
	BBB permeability	−1.101	Numeric (log BB)
	CNS permeability	−3.515	Numeric (log PS)
**Metabolism**	CYP2D6 substrate	No	Categorical (Yes/No)
	CYP3A4 substrate	Yes	Categorical (Yes/No)
	CYP1A2 inhibitior	No	Categorical (Yes/No)
	CYP2C19 inhibitior	No	Categorical (Yes/No)
	CYP2C9 inhibitior	No	Categorical (Yes/No)
	CYP2D6 inhibitior	No	Categorical (Yes/No)
	CYP3A4 inhibitior	No	Categorical (Yes/No)
**Excretion**	Total Clearance	0.404	Numeric (log mL/min/kg)
	Renal OCT2 substrate	No	Categorical (Yes/No)
**Toxicity**	AMES toxicity	No	Categorical (Yes/No)
	Max. tolerated dose (human)	0.25	Numeric (log mg/kg/day)
	hERG I inhibitor	No	Categorical (Yes/No)
	hERG II inhibitor	Yes	Categorical (Yes/No)
	Oral Rat Acute Toxicity (LD_50_)	2.554	Numeric (mol/kg)
	Oral Rat Chronic Toxicity (LOAEL)	3.51	Numeric (log mg/kg_bw/day)
	Hepatotoxicity	No	Categorical (Yes/No)
	Skin Sensitisation	No	Categorical (Yes/No)
	*T. pyriformis* toxicity	0.286	Numeric (log ug/L)
	Minnow toxicity	0.168	Numeric (log mM)

**Table 3:** Gene Ontology (GO) enrichment analysis of targets common to empagliflozin and CKD across Biological Process, Cellular Component, and Molecular Function categories.

**GO Biological Process**				
**Enrichment FDR**	**nGenes**	**Pathway Genes**	**Fold Enrichment**	**Pathway**
4.34 × 10^−30^	84	1994	4.441906364	GO:0016310 phosphorylation
9.19 × 10^−29^	76	1684	4.758682969	GO:0006468 protein phosphorylation
1.24 × 10^−28^	77	1752	4.634169244	GO:1901700 response to oxygen-containing compound
4.82 × 10^−27^	73	1660	4.636924657	GO:0009719 response to endogenous stimulus
4.82 × 10^−27^	60	1061	5.962812233	GO:0010243 response to organonitrogen compound
1.25 × 10^−26^	62	1172	5.578010726	GO:1901698 response to nitrogen compound
4.31 × 10^−24^	54	956	5.955951256	GO:0051338 reg. of transferase activity
3.28 × 10^−23^	63	1409	4.714599693	GO:0071495 cellular response to endogenous stimulus
1.20 × 10^−22^	72	1916	3.962344747	GO:0042592 homeostatic proc.
1.25 × 10^−22^	49	826	6.255057408	GO:0043549 reg. of kinase activity
2.03 × 10^−22^	59	1272	4.890802974	GO:1901701 cellular response to oxygen-containing compound
1.29 × 10^−21^	51	958	5.613321724	GO:0014070 response to organic cyclic compound
8.97 × 10^−21^	59	1375	4.524437369	GO:0018193 peptidyl-amino acid modification
3.83 × 10^−20^	57	1318	4.560103634	GO:0042325 reg. of phosphorylation
4.86 × 10^−20^	60	1478	4.280476170	GO:0019220 reg. of phosphate metabolic proc.
4.86 × 10^−20^	60	1479	4.277582001	GO:0051174 reg. of phosphorus metabolic proc.
5.39 × 10^−20^	63	1641	4.048062747	GO:0031399 reg. of protein modification proc.
6.83 × 10^−19^	65	1836	3.732982440	GO:0023056 positive reg. of signaling
1.12 × 10^−18^	42	734	6.033488617	GO:1901699 cellular response to nitrogen compound
1.32 × 10^−18^	39	622	6.611339962	GO:0051347 positive reg. of transferase activity
**GO Cellular Component**				
**Enrichment FDR**	**nGenes**	**Pathway Genes**	**Fold Enrichment**	**Pathway**
6.32 × 10^−16^	62	1894	3.451651833	GO:0005887 integral component of plasma membrane
2.64 × 10^−15^	62	1978	3.305070056	GO:0031226 intrinsic component of plasma membrane
1.51 × 10^−11^	12	54	23.43164363	GO:0000307 cyclin-dependent protein kinase holoenzyme complex
9.05 × 10^−9^	40	1332	3.166438328	GO:0098590 plasma membrane region
1.73 × 10^−8^	14	150	9.841290323	GO:1902911 protein kinase complex
2.84 × 10^−8^	34	1050	3.414325214	GO:0009986 cell surface
2.87 × 10^−8^	13	132	10.38447842	GO:1902554 serine/threonine protein kinase complex
1.41 × 10^−7^	39	1435	2.865681851	GO:0045202 synapse
1.70 × 10^−7^	25	653	4.036845188	GO:0030425 dendrite
1.70 × 10^−7^	25	655	4.024518943	GO:0097447 dendritic tree
1.70 × 10^−7^	15	223	7.092537869	GO:0101002 ficolin-1-rich granule
2.25 × 10^−7^	20	429	4.915729432	GO:0043235 receptor complex
2.48 × 10^−7^	29	888	3.443501681	GO:0036477 somatodendritic compartment
2.60 × 10^−7^	18	351	5.407302375	GO:0045121 membrane raft
2.60 × 10^−7^	18	352	5.391940721	GO:0098857 membrane microdomain
3.46 × 10^−7^	12	142	8.910625041	GO:1904813 ficolin-1-rich granule lumen
1.34 × 10^−6^	18	395	4.804969959	GO:0097060 synaptic membrane
2.87 × 10^−6^	20	513	4.110814671	GO:0043025 neuronal cell body
3.22 × 10^−6^	16	332	5.081561268	GO:0061695 transferase complex transferring phosphorus-containing groups
3.60 × 10^−6^	10	115	9.168904027	GO:0043202 lysosomal lumen
**GO Molecular Function**				
**Enrichment FDR**	**nGenes**	**Pathway Genes**	**Fold Enrichment**	**Pathway**
2.27 × 10^−23^	48	748	6.766356983	GO:0016773 phosphotransferase activity alcohol group as acceptor
4.32 × 10^−23^	50	849	6.209799547	GO:0016301 kinase activity
4.53 × 10^−23^	44	632	7.340926326	GO:0004672 protein kinase activity
4.86 × 10^−22^	38	470	8.525129915	GO:0004712 protein serine/threonine/tyrosine kinase activity
3.57 × 10^−20^	50	1008	5.230277595	GO:0016772 transferase activity transferring phosphorus-containing groups
1.41 × 10^−18^	61	1662	3.870027783	GO:0005524 ATP binding
8.66 × 10^−18^	61	1729	3.720061408	GO:0032559 adenyl ribonucleotide binding
1.07 × 10^−17^	61	1741	3.694420549	GO:0030554 adenyl nucleotide binding
4.85 × 10^−14^	38	824	4.862634781	GO:0019900 kinase binding
1.19 × 10^−13^	29	471	6.492206992	GO:0004674 protein serine/threonine kinase activity
1.12 × 10^−12^	18	159	11.93687505	GO:0004713 protein tyrosine kinase activity
1.63 × 10^−12^	34	739	4.851206326	GO:0019901 protein kinase binding
1.39 × 10^−11^	24	376	6.730365722	GO:0106310 protein serine kinase activity
3.63 × 10^−11^	53	1908	2.928955453	GO:0038023 signaling receptor activity
3.63 × 10^−11^	53	1908	2.928955453	GO:0060089 molecular transducer activity
9.77 × 10^−11^	48	1646	3.074869394	GO:0004888 transmembrane signaling receptor activity
3.88 × 10^−8^	16	231	7.303369442	GO:0017171 serine hydrolase activity
5.07 × 10^−8^	6	13	48.66572138	GO:0008353 RNA polymerase II CTD heptapeptide repeat kinase activity
5.07 × 10^−8^	11	91	12.74578417	GO:0051219 phosphoprotein binding
2.16 × 10^−7^	15	227	6.967559228	GO:0008236 serine-type peptidase activity

**Table 4:** KEGG pathways associated with shared molecular targets of empagliflozin and CKD (enrichment profiles retrieved from KEGG database: https://www.kegg.jp/kegg/kegg1.html; accessed on 6 February 2026).

Enrichment FDR	nGenes	Pathway Genes	Fold Enrichment	Pathway
4.12 × 10^−23^	41	530	8.156864620	hsa05200 Pathways in cancer
8.87 × 10^−21^	21	97	22.82773528	hsa05215 Prostate cancer
4.73 × 10^−19^	31	354	9.233656174	hsa04151 PI3K-Akt signaling pathway
8.25 × 10^−17^	29	362	8.447042799	hsa04080 Neuroactive ligand-receptor interaction
1.92 × 10^−16^	18	102	18.60748170	hsa04914 Progesterone-mediated oocyte maturation
1.92 × 10^−16^	24	232	10.90783410	hsa05171 Coronavirus disease-COVID-19
3.30 × 10^−16^	24	240	10.54423963	hsa04020 Calcium signaling pathway
7.77 × 10^−16^	19	131	15.29317198	hsa04068 FoxO signaling pathway
1.17 × 10^−15^	20	156	13.51825594	hsa04218 Cellular senescence
2.24 × 10^−15^	20	162	13.01757979	hsa05161 Hepatitis B
2.86 × 10^−15^	15	70	22.59479921	hsa05230 Central carbon metabolism in cancer
2.86 × 10^−15^	17	103	17.40311395	hsa04660 T cell receptor signaling pathway
6.83 × 10^−15^	17	109	16.44514438	hsa04066 HIF-1 signaling pathway
9.19 × 10^−15^	15	76	20.81099927	hsa05212 Pancreatic cancer
1.78 × 10^−14^	18	137	13.85374550	hsa05135 Yersinia infection
1.99 × 10^−14^	19	161	12.44351261	hsa05206 MicroRNAs in cancer
2.46 × 10^−14^	21	214	10.34715104	hsa05417 Lipid and atherosclerosis
2.47 × 10^−14^	16	100	16.87078341	hsa04933 AGE-RAGE signaling pathway in diabetic complications
4.62 × 10^−14^	21	222	9.974280732	hsa05166 Human T-cell leukemia virus 1 infection
7.87 × 10^−14^	16	108	15.62109575	hsa04659 Th17 cell differentiation

## Data Availability

All data generated or analyzed during this study are included in this published article.

## Abbreviations

The following abbreviations are used in this manuscript:

ADMET	Absorption, Distribution, Metabolism, Excretion, and Toxicity
AGE(s)	Advanced glycation end product(s)
ADT	AutoDock Tools
AKT	Protein kinase B (Akt)
AMES	Ames mutagenicity test
CKD	Chronic kidney disease
CNS	Central nervous system
DKD	Diabetic kidney disease
EGFR	Epidermal growth factor receptor
EMT	Epithelial–mesenchymal transition
ERK	Extracellular signal-regulated kinase
ESRD	End-stage renal disease
GAPDH	Glyceraldehyde-3-phosphate dehydrogenase
GO	Gene ontology
HSP90AA1	Heat shock protein 90 alpha family class A member 1
HSP90AB1	Heat shock protein 90 alpha family class B member 1
HTLV-1	Human T-lymphotropic virus 1
KEGG	Kyoto Encyclopedia of Genes and Genomes
MAPK	Mitogen-activated protein kinase
mTOR	Mechanistic target of rapamycin
STRING	Search Tool for the Retrieval of Interacting Genes/Proteins
UniProt	Universal Protein Resource
